# Competing Mechanisms of Gamma and Beta Oscillations in the Olfactory Bulb Based on Multimodal Inhibition of Mitral Cells Over a Respiratory Cycle^1,2,3^

**DOI:** 10.1523/ENEURO.0018-15.2015

**Published:** 2015-12-08

**Authors:** François David, Emmanuelle Courtiol, Nathalie Buonviso, Nicolas Fourcaud-Trocmé

**Affiliations:** 1 Lyon Neuroscience Research Center, CNRS UMR 5292, INSERM U1028, Université Claude Bernard, F-69372 Lyon, France; 2 Emotional Brain Institute, Nathan Kline Institute for Psychiatric Research and the Department of Child and Adolescent Psychiatry, New York University Langone Medical Center, New York, New York 10022

**Keywords:** Beta oscillations, computational model, gamma oscillations, sensory processing, short-term plasticity, top-down processing

## Abstract

Gamma (∼40-90 Hz) and beta (∼15-40 Hz) oscillations and their associated neuronal assemblies are key features of neuronal sensory processing. However, the mechanisms involved in either their interaction and/or the switch between these different regimes in most sensory systems remain misunderstood. Based on *in vivo* recordings and biophysical modeling of the mammalian olfactory bulb (OB), we propose a general scheme where OB internal dynamics can sustain two distinct dynamic states, each dominated by either a gamma or a beta regime. The occurrence of each regime depends on the excitability level of granule cells, the main OB interneurons. Using this model framework, we demonstrate how the balance between sensory and centrifugal input can control the switch between the two oscillatory dynamic states. In parallel, we experimentally observed that sensory and centrifugal inputs to the rat OB could both be modulated by the respiration of the animal (2-12 Hz) and each one phase shifted with the other. Implementing this phase shift in our model resulted in the appearance of the alternation between gamma and beta rhythms within a single respiratory cycle, as in our experimental results under urethane anesthesia. Our theoretical framework can also account for the oscillatory frequency response, depending on the odor intensity, the odor valence, and the animal sniffing strategy observed under various conditions including animal freely-moving. Importantly, the results of the present model can form a basis to understand how fast rhythms could be controlled by the slower sensory and centrifugal modulations linked to the respiration.

Visual Abstract: See Abstract

## Abstract (precis)

Gamma (∼40-90 Hz) and beta (∼15-40 Hz) oscillations and their associated neuronal assemblies are key features of neuronal sensory processing. However, the mechanisms involved in either their interaction and/or the switch between these different regimes in most sensory systems remain misunderstood.

## Visual Abstract

**Figure:**
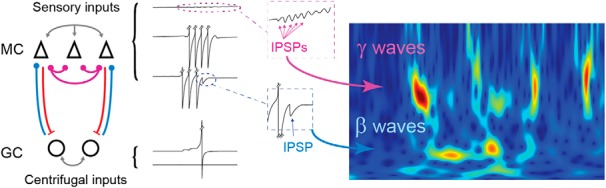

## Significance Statement

Neuronal oscillations accompany the sensory perception at multiple timescales. Fast-paced activities (gamma, ∼40-90 Hz; beta, ∼15-40 Hz) facilitate discrimination and signal cognitive response. Slower processes (2-12 Hz) gate the time window for sensory and centrifugal inputs to ascend and descend, respectively, relative to sensory relays. In the olfactory bulb, which is the first relay of the olfactory system, the main local interneurons provide a major interface between ascending and descending activities. The balance between these two pathways controls the two types of inhibition released by these interneurons on the main relay cells and thereby the network oscillatory dynamics. Using minimalist computational simulations and *in vivo* experiments, we proposed a general scheme intimately linked to olfactory processing.

## Introduction

Oscillatory activity underlying neuronal assembly formation is crucial in most functions, including environment perception, adaptive motor responses, and memory formation (Engel and Singer, 2001; Tallon-Baudry et al., 2001; Varela et al., 2001). Those oscillatory mechanisms involve fine and broader timescales (Schroeder et al., 2010). Due to interarea connections, these mechanisms proved to be difficult to disentangle. In this regard, the olfactory bulb (OB) is well suited to investigate those mechanisms because of both the ability to handle separately the arrival of sensory inputs and centrifugal fibers (Ravel and Pager, 1990; Boyd et al., 2012; Markopoulos et al., 2012), and the prominence of these multiscale temporal phenomena.

The OB expresses two fast dichotomous regimes, beta (15-40 Hz) and gamma (40-90 Hz), which can be subdivided into two bands in particular conditions (Kay, 2003; Manabe and Mori, 2013), and a slower one (in the theta frequency range 2-12 Hz) related to respiratory rhythm (for review see, Kay, 2014; Martin and Ravel, 2014). On a functional level, gamma oscillations are linked to odor quality (Kashiwadani et al., 1999; Cenier et al., 2008), odor intensity (Neville and Haberly, 2003; Courtiol et al., 2011a), and odor learning (Kay, 2014; Martin and Ravel, 2014). Beta oscillations are observed in response to particular odorants (Chapman et al., 1998; Zibrowski et al., 1998), reflecting pure sensory-processing dynamics but also depend strongly on the experience (Martin et al., 2004; Kay, 2014). Importantly, the occurrence pattern of those two fast alternating oscillations are intertwined with the respiratory slow rhythm (Buonviso et al., 2003; Lepousez and Lledo, 2013; Fukunaga et al., 2014), which provides a window for odor discrimination (Uchida et al., 2006; Bathellier et al., 2008a; Shusterman et al., 2011). Gamma oscillations tend to appear locked to the inspiration–expiration transition (Manabe and Mori, 2013), whereas beta oscillations can either be locked to the late expiration (Buonviso et al., 2003; Cenier et al., 2008) or cover multiple respiratory cycles in awake conditions. While it is known that fast gamma and beta rhythms both rely on the dendrodendritic interaction between excitatory mitral cells (MCs)/tuft cells (TCs) and inhibitory granule cells (GCs; Rall and Shepherd, 1968; Lagier et al., 2004; Manabe and Mori, 2013; Fourcaud-Trocmé et al., 2014; Fukunaga et al., 2014; Lepousez et al., 2014), with the expression of beta oscillations requiring the integrity of the relation OB-cortex (Neville and Haberly, 2003; Martin et al., 2006), the mechanisms that control the beta–gamma switch are not well understood. Here, we proposed to test the hypothesis that the mechanistic process underlying the beta–gamma switch (which could be clear cut or more graded) relies on a competition between the balanced influence of sensory and centrifugal inputs. We approached this question through a simple biophysical model complemented by experimental observations in various conditions to calibrate the model outputs. This model aims to give plausible mechanisms able to explain (1) the generation of both gamma and beta rhythms by the same network of excitatory and inhibitory neurons, (2) what controls the switch between both, and (3) the phase relationship between fast oscillations and the respiratory cycle, as this is crucial for sensory and multisensory integration (Deschênes et al., 2012). More precisely, the generic mechanisms present in the model are as follows: (1) an entrainment mechanism for the gamma oscillations (Wang, 2010); (2) a separate mechanism for the emergence of beta oscillations based on a GC spike-dependent PING (pyramidal-interneuron gamma) mechanism (Börgers and Kopell, 2003; Brea et al., 2009; Fourcaud-Trocmé et al., 2011); and (3) a slow respiration-like modulation of both sensory and top-down inputs based on known and new experimental data. The simulations showed that GC activation mode (local via peripheral inputs vs global via centrifugal inputs) determines the dominant frequency of the oscillatory regime (gamma or beta, respectively). The switch to another regime depends critically on the balance and the relative timing between sensory and centrifugal inputs.

Based on a minimal set of experimentally well described elements, this model provides a mechanistic basis to understand the different dynamic states of the bulbar network related to sensory processing and their interaction during various behavioral conditions. It also sets the dynamic framework for understanding how additional neuronal components (Batista-Brito et al., 2008; Eyre et al., 2008; Fukunaga et al., 2012; Manabe and Mori, 2013; Miyamichi et al., 2013) could enrich the dynamics that are necessary for a proper olfactory performance.

## Materials and Methods

### Experiments

#### Preparation and recordings

All animal procedures were performed in accordance with the regulations of the authors’ institutional animal care committee. All efforts were made to minimize animal suffering and the number of animals used. Experiments were performed on male adult Wistar rats (260–400 g; Charles River Laboratories), which were maintained on a normal diet and under a 6:00 A.M. to 6:00 P.M. lights-on regimen.

Rats were anesthetized with urethane (1.5 mg/kg, i.p.; with additional supplements as needed) and placed in a stereotaxic apparatus. The dorsal region of the OB was exposed. Bulbar activity was recorded as a broadband signal (0.1-5 kHz) using linear 16-channel silicon probes (NeuroNexus Technologies) with a home-made, 16-channel DC amplifier. Electrodes on the probe were spaced by 50 μm. The data were digitally sampled at 10 kHz and acquired on a PC using the IOTech acquisition system (Wavebook, IOTech Inc.). Lateral olfactory tract (LOT) electrical stimulations were performed via bipolar stainless steel electrodes, which were stereotaxically positioned in the LOT (bregma coordinates: anteroposterior, 3.7 mm; lateral, 3.4 mm). Optimal placement was determined by the observation of field potentials evoked in the OB by electrical stimulation (constant current square pulses, 100 µs; amplitude range, 0.1-0.5 mA). The respiration frequency was used to monitor the depth of anesthesia, and the injection of urethane was performed accordingly when the frequency increased.

The respiration signal was recorded using a homemade flowmeter based on a fast response time thermodilution airflow sensor (bidirectional microbridge mass airflow sensor, AWM 2000 family, Micro Switch Honeywell; described in detail in the study by Roux et al., 2006). The respiratory phases of the LOT-evoked potential and other events were computed by detecting five landmarks on the respiratory signal [the inspiration (I) maximum, the I/expiration (I/E) transition, the E maximum, the E plateau (EP), and the E/I transition)]. We then aligned landmarks of different respiratory cycles and used a linear phase advance between two landmarks.

Odors were delivered at 9% of the saturated vapor pressure (SVP) during 5 s through a dilution olfactometer (440 ml/min). Odors used were 2-heptanone, ethyl-benzoate, heptanal, and isoamyl-acetate. The odorant stimulation does not aim to simulate natural stimuli, as those probably activate more glomeruli at a lower concentration (Vincis et al., 2012), but rather to present a simple and reliable way to elicit gamma and beta oscillations similar to those observed in natural conditions in freely behaving animals (i.e., to have a physiological model of fast oscillation). In anesthetized conditions, the high concentration induces oscillation amplitudes similar to the ones observed in awake conditions or in response to low-concentration stimulation (Rosero and Aylwin, 2011; Lepousez et al., 2014). No aversive behavior of the animals was observed in our group for those odorants at that concentration (our unpublished observation). Depending on the experiment, rats were either freely breathing or tracheotomized in order to control for the intensity of the airflow input. In the latter case, odors were either simply pulled continuously into the nasal cavity through constant aspiration or were delivered using a rhythmic nasal airflow reproducing breathing dynamics but with different amplitudes.

#### CSD

Local field potentials (LFPs) in the OB were recorded with linear 16-channel silicon probes (a1 × 16-5 mm 50-177, NeuroNexus) inserted perpendicularly into OB layers. A one-dimensional current source density (CSD) analysis (for review of CSD, see Mitzdorf, 1985) was performed using the inverse CSD method (Pettersen et al., 2006). For each recording location, the electrode closest to the MC layer (MCL) was determined off-line by searching for the flattest LFP response to LOT electrical stimulation (Rall and Shepherd, 1968). Channels in the glomerular layer (GL), external plexiform layer (EPL), and GC layer (GCL) were identified according to their distance to the MCL. Finally, CSD maps were averaged across recording sessions (*n* = 18 animals or electrode insertions; 2 animals had 2 insertions, others had only 1) after spatial alignment on their MCL. Practically, the EPL current source amplitude was computed as the median of the 20% highest points, from any of the three electrodes immediately more superficial than the MCL, in the time window of interest (10-60 ms following the peak of the LOT-evoked LFP response). Source amplitudes from a given recording location were normalized to a mean of 1 in order to compare changes along the respiratory cycles in different recording sessions and locations.

#### Multiunit activity

Multiunit activity data was obtained by high-pass filtering the OB signals >300 Hz of the three electrodes closest to the MCL (for the 18 recorded session locations). Spikes were detected as peaks larger than 7 times the SD of the filtered signal and with a minimum interspike interval of 2 ms.

### Simulations

#### Neuron model characteristics

##### Mitral cell model

**Figure 1.**
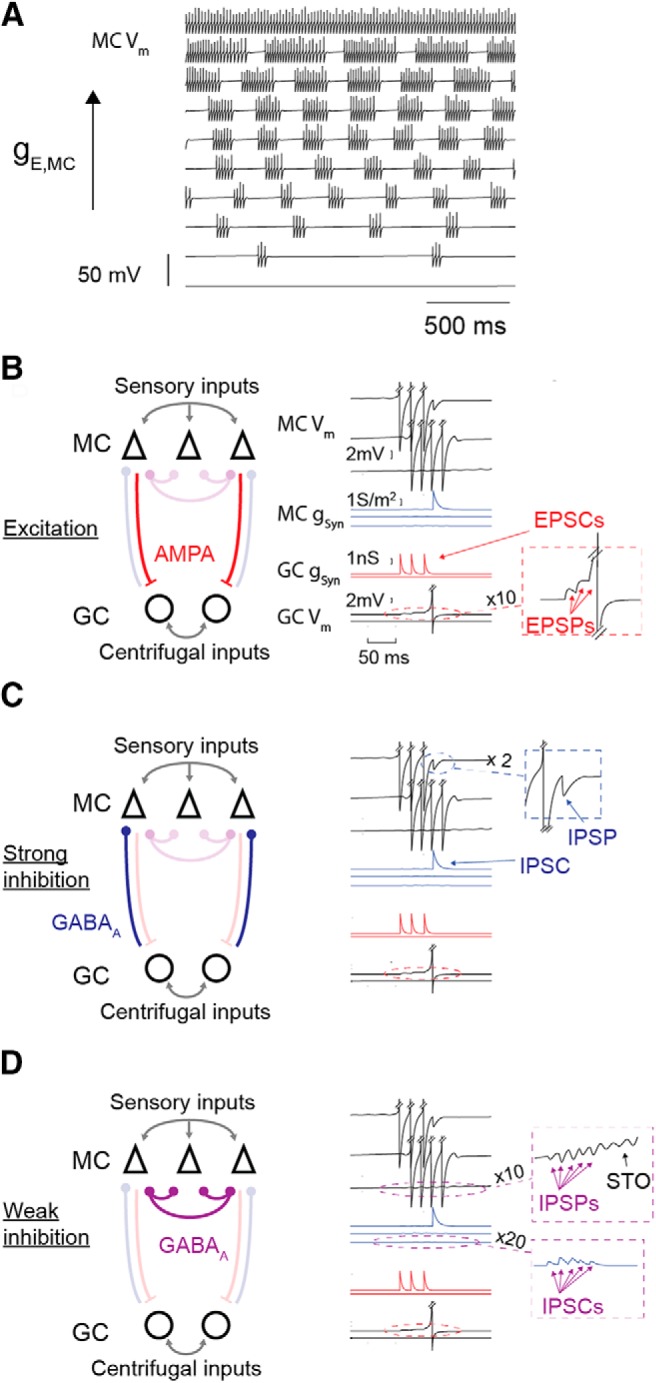
Synaptic connections of the network. ***A***, MC intrinsic responses to a range of excitatory conductances (*g_E_*). ***B***, Network properties. Excitatory connections consist of AMPA synapses (red) from MC to GC. They are activated by MC presynaptic spikes and can be subject to short-term plasticity used later on. They generate EPSCs (red trace) specific to each GC. EPSPs (see inset) are integrated at the GC soma and can trigger a spike if the resting membrane potential of the GC is depolarized enough (especially by centrifugal modulation). ***C***, Inhibitory connections consist of GABA_A_ synapses, which are activated by presynaptic GC spikes (strong inhibition). They generate much larger IPSPs (see inset) than GC spike-independent inhibition. ***D***, Inhibitory connections also consist of GABA_A_ synapses (purple), which are activated by MC presynaptic spikes corresponding to local integration of the GC spines without depending on GC spikes (weak inhibition). They generate IPSCs (see bottom inset) in all MCs with distinct delays. The IPSPs (see top inset) can be followed by intrinsic subthreshold oscillations (STOs). ***C***, Note that a full network contains 100 MCs and 100 GCs, MCs are all-to-all coupled via weak inhibition, and any MC–GC pair is reciprocally connected with AMPA and strong inhibition with a probability of 0.5.

The MC model used was adapted from previous studies (Wang and Buzsáki, 1996; Bathellier et al., 2006; David et al., 2009). The model uses parameters from Bhalla and Bower (1993) and Wang (1993). Its essential features are (1) its spiking activity through sodium spikes, (2) its bursting activity, (3) its current frequency response, (4) its resonant properties as revealed through subthreshold oscillations, and (5) its phase response curve, as described into detail in the study by David et al. (2009). An example of the responses of the model to a range of excitatory conductances is presented in Figure 1*A*.

The variables of the model are the membrane potential (*V_m_*), the activation gating variable of the fast rectifying potassium current (*m_Kf_*), the activation (*m_Ks_*), and the inactivation (*h_Ks_*) gating variables of the slow potassium current. The Equations 1–4 fully describe its dynamic behavior, as follows:

(Eq. 1) $$\begin{array}{l} C\frac{dV_{m}}{dt}=-g_{L}(V_{m}-E_{L})-g_{Na}m_{Na,\infty }^{\text{3}}(V_{m}-E_{Na})\\ \;-g_{NaP}m_{NaP,\infty }(V_{m}-E_{Na})-g_{Kf}m_{Kf}(V_{m}-E_{K})\\ \;-g_{KA}{\overset{¯}{m}}_{KA}{\overset{¯}{h}}_{KA}(V_{m}-E_{K})-g_{Ks}m_{Ks}h_{Ks}(V_{m}-E_{K})\\ \;+I_{syn,I}+I_{input,E} \end{array}$$

(Eq. 2) $$\frac{dm_{Kf}}{dt}=-\frac{m_{Kf}}{\tau_{Kf}^{m}}$$

(Eq. 3) $$\frac{dm_{Ks}}{dt}=\frac{m_{Ks,\infty }-m_{Ks}}{\tau_{Ks}^{m}}$$

(Eq. 4) $$\frac{dh_{Ks}}{dt}=\frac{h_{Ks,\infty }^{}-h_{Ks}}{\tau_{Ks}^{h}}$$

The gating variable parameters were given by the following:

$$m_{\infty ,Na}=\frac{a_{Na}^{m}}{a_{Na}^{m}+b_{Na}^{m}}$$

$$a_{Na}^{m}=\text{0}\text{.32}\cdot \frac{V_{m}+\text{50}}{\text{1-exp}(-\frac{V_{m}+\text{50}}{\text{4}})}$$

$$b_{Na}^{m}=\text{0}\text{.28}\cdot \frac{V_{m}+\text{23}}{\text{exp}(\frac{V_{m}+\text{23}}{\text{5}})-\text{1}}$$

$$m_{\infty ,Nap}=\frac{\text{1}}{\text{exp}(-\frac{V_{m}+\text{51}}{\text{5}})+\text{1}}$$

$$m_{\infty ,Ks}=\frac{\text{1}}{\text{1}+\text{exp}(-\frac{V_{m}+\text{34}}{\text{6}\text{.5}})}$$

$$h_{\infty ,Ks}=\frac{\text{1}}{\text{1}+\text{exp}(\frac{V_{m}+\text{65}}{\text{6}\text{.6}})}$$

$$\tau_{Ks}^{h}=1\text{00}+\frac{11\text{0}}{\text{exp}(-\frac{V_{m}+\text{71}\text{.6}}{\text{6}\text{.85}})+\text{1}}$$

$$\tau_{Kf}^{m}=\;2.6\,\;ms$$

$$\tau_{Ks}^{m}=\;10\,\;ms\text{.}$$

Each time the membrane potential reached −30 mV, a spike was generated, *V_m_* was reset to −65 mV, *m_Ks_* was incremented by 0.03, *h_Ks_* was incremented by 0.002, and *m_Kf_* was incremented by 0.4 in order to reproduce the dynamics of the membrane potential after each spike and to preserve the transition from spiking to subthreshold oscillations as observed in the studies by Desmaisons et al. (1999) and Balu et al. (2004).

The maximal conductance of the ionic channels was *g_Na_* = 500 S/m^2^; *g_NaP_* = 1.1 S/m^2^; *g_Ks_* = 310 S/m^2^; *g_KA_* = 100 S/m^2^; *g_Kf_* = 100 S/m^2^. The leak conductance is *g_L_* = 0.1 S/m^2^. The product ${\bar{m}}_{KA}\cdot {\bar{h}}_{KA}$ is approximated to 0.004. The reverse ionic potentials are *E_K_* = −75 mV and *E_Na_* = 45 mV, and the membrane reversal potential is E_L_ = −66.5 mV. The membrane capacitance was set to 0.01 F/m^2^.

##### Granule cell model

We used a quadratic integrate and fire (QIF) model (Börgers and Kopell, 2003) for which parameters were fitted to reproduce the frequency–current relationship observed in a more detailed model of GCs (Davison et al., 2003). Its single variable, *V_m_*, was described by Equation 5, as follows:

(Eq. 5) $$\tau_{m}\frac{dV_{m}}{dt}=\frac{{\left(V_{m}-V_{T}\right)}^{2}}{2\Delta_{T}}-\frac{I_{T}}{g_{L}}+\frac{I_{centrifugal,E}+I_{syn,E}}{g_{L}}$$

Each time the membrane potential reached the threshold of 0 mV; it was reset to −70 mV. The membrane time constant for the granule cell was set to τ_m_ = 60 ms (Schoppa et al., 1998). The QIF threshold *V_T_* = −60 mV was based on the spike shape observed in the detailed GC model (Davison et al., 2003). *g_L_* =16.66 nS, Δ*_T_* = 0.1 mV, *I_T_* = 0.02 nA were parameters that resulted from the fit.

#### Synaptic inputs at the dendrodendritic synaptic junction

All phasic synaptic currents were described by an equation (Eq.6) of the form:

(Eq.6) $$I_{syn,X}=-g_{X,\max}s_{X}(V-E_{X})$$

where *X* is the considered current, *g_X_*_,max_ is the maximal synaptic conductance, *s_X_* is the synaptic activation (fraction of open channels between 0 and 1), and *E_X_* is the synaptic reversal potential. Parameter values per synapse are given in the following sections.

##### AMPA synapses

The fraction of open channels of AMPA synapses from MCs to GCs (Fig. 1*B*) was modeled as a single exponential described by Equation 7, as follows:

(Eq.7) $$\frac{ds_{E}}{dt}=-\frac{s_{E}}{\tau_{E}}.$$

The decay time constant τ*_E_* was set to 3 ms according to Schoppa et al. (1998). A synaptic delay of 1 ms was set from the spike time of the MC to the start of the EPSC (Davison et al., 2003). Other parameters were *E_E_* = 0 mV and *g_E_*_,max_ = 4 nS.

##### GABA_A_ synapses

The GABA_A_ synaptic current (*E_I_* = −70 mV) received by MCs had the following three components: (1) the tonic part, consisting of the constant inhibitory current, received by all MCs, of conductance *g_I,cst_* = 20 S/m^2^ (David et al., 2009) as it is present in most of MCs during odor presentation (Yokoi et al., 1995; Kollo et al., 2014); (2) GC spike-dependent GABA_A_ synapses, a phasic conductance *s_I,G_ g_I_*_,_*_G_*_,max_ triggered by GC spikes (Fig. 1*C*), with the fraction of open channels modeled as follows as a simple exponential described by Equation 8, as follows:

(Eq. 8) $$\frac{ds_{I,G}}{dt}=-\frac{s_{I,G}}{\tau_{I,G}}$$

The decay time constant was set to τ*_I,G_* = 7 ms corresponding to ranges experimentally observed for mini-IPSC measurement (Castillo et al., 1999; Bathellier et al., 2006; Lagier et al., 2007; Eyre et al., 2012). The maximum conductance of a single synaptic input *g_I,G_*_,max_= 3 S/m^2^ has been estimated to be in the range of experimental data (current amplitude, 20 pA; Schoppa et al., 1998). (3) GC spike-independent GABA_A_ synapses, consisting of a phasic conductance *s_I_ g_I_*_,max_ corresponding to a dendrodendritic inhibition, independent of granule spiking, directly triggered by a mitral spike (Fig. 1*D*; *g_I,_*_max_ = 0.18 S/m^2^). The fraction of open channels was modeled as a double exponential described by Equations 9 and 10, as follows:

(Eq.9) $$\frac{ds_{I}}{dt}=\frac{r_{I}-s_{I}}{\tau_{I}}$$

(Eq. 10) $$\frac{dr_{I}}{dt}=-\frac{r_{I}}{\tau_{r}}$$

The rise time constant τ*_r_* was set to 2 ms, and the decay time constant was set to τ*_I_* = 7 ms, adapted from estimated values (Margrie and Schaefer, 2003; Schoppa, 2006). A synaptic delay was randomly chosen from a uniform distribution between 5 and 13 ms from the spike time of the MC to the start of the IPSC. This aimed to account for both (1) the average relative timing seen between MC spikes and inhibitory events (Lagier et al., 2004) due to the two synapses MC–GC and GC–MC being involved in this connection and (2) a less reliable way of inhibition allowing weak coupling in the network.

In order to reflect the partial activation of the GC synaptic spines, GC spike-independent GABA conductance was weak compared with the GC spike-dependent GABA conductance (Egger et al., 2005).

##### Short-term plasticity

Short-term plasticity was introduced to reproduce fast-adapting AMPA synapses from MC to GC (Balu et al., 2007). The maximal conductance decays with time according to the formalism of the following equations (Eqs. 11, 12) introduced by Markram et al. (1998):

(Eq.11) $$\frac{dx}{dt}=\frac{1-x}{\tau_{d}}$$

(Eq.12) $$\frac{du}{dt}=\frac{U-u}{\tau_{r}}$$

The parameters were set to τ*_d_* = 150 ms, τ_r_ = 1 ms, and *U* = 1 in order to have a pure depressing synapse without facilitation. Each time a presynaptic spike is emitted, this triggers a change in the values of *x* and *u*. *u* takes the value *u* + *U*(1 − *u*), and *x* takes the value *x*(1 − *u*). The synaptic weights were modulated by the product *x* * *u* as implemented in the Brian simulator (Goodman and Brette, 2009). When short-term plasticity was introduced (starting at Fig. 4 and later), we used *g_E_*_,max_ = 2.5 * *g_E_*_,max,default_ = 1 nS.

##### Connectivity

The 100 MCs and 100 GCs were connected for all the present simulations. Due to the long range of mitral lateral dendrites, we first assumed that any pair of mitral cells was connected through a granule cell (Xiong and Chen, 2002). In the model, this was accounted for by an all-to-all coupling between MCs through GC spike-independent inhibition. Second, we assumed that any MC–GC pair has a 0.5 chance to form a dendrodendritic synapse with both an AMPA synaptic connection from MC to GC and a GC spike-dependent GABA synaptic connection from GC to MC. Changes in cell numbers or synapse density were not found to affect critically the tendency of the results. Unless mentioned otherwise the connectivity was randomly drawn for each simulation each time we used groups of multiple simulations.

#### Sensory and centrifugal inputs

##### Sensory inputs modulation

Sensory inputs were modeled as an excitatory conductance (*E*_input,_*_E_* = 0 mV) assigned to each MC. For constant input, *g*_input,_*_E_* values were linearly spaced across MCs between 6.1 and 7.6 S/m^2^. When a respiration-like sampling was introduced, unless otherwise mentioned, these excitatory conductances were modulated from a basal value common for all MCs (*g*_input,_*_E_*_,basal_ = 4 S/m^2^) to a maximum value *g*_input,_*_E_*_,max_ linearly spaced across mitral cells between 6.6 and 8.1 S/m^2^. A phase shift between each MC was introduced so as to obtain a delay of response observed in MCs corresponding to observations across several species at both the neuroreceptor and the MC levels (Cang and Isaacson, 2003; Spors et al., 2006; Schaefer and Margrie, 2007; Rospars et al., 2013). The variability of the delay of response can reflect odor concentration, odor identity, or nasal flow rate.

The general equation of the sensory modulation of MCs is described in Equation 13, for MC *i*, as follows:

(Eq.13) $$g_{input,E,i}\left(t\right)=\frac{g_{input,E,basal}+g_{injput,E,\max,i}}{2}+\frac{g_{injput,E,\max,i}-g_{input,E,basal}}{2}\left[1+\cos\left(2\pi \cdot f\cdot t+\phi_{i}\right)\right]$$

where *t* is the time*, f* is the frequency of sensory modulation (from 2 Hz in anesthesia to 12 Hz during sniffing), φ*_i_* is the phase shift specific for each MC (see next paragraph for the determination of phase shift values). The maximum of the sine wave without phase shift corresponds approximately to the inspiration–expiration transition.

##### Odor intensity variation

In this study, we chose to simulate changes in sensory input strength by a modulation of MC locking relative to the respiratory cycle. This takes into account the existence of (1) a normalization process at the interglomerular level (Cleland et al., 2007) and an all-or-nothing response at the intraglomerular level (Gire and Schoppa, 2009), which indicate that the level of excitability of MCs could be relatively stable with odor intensity; (2) a general decrease of latency in the bulbar response (Spors et al., 2006; Zhou and Belluscio, 2012; Rospars et al., 2013; Yu et al., 2013); and (3) a better locking of MC to the respiration with high odor intensity (Courtiol et al., 2011a). These are only part of the many parameters that account for the adaptation of the MC response to the odor intensity (Yokoi et al., 1995; Chalansonnet and Chaput, 1998; Yu et al., 2013; Migliore et al., 2014; Gupta et al., 2015).

To simulate a change of odor intensity, the phase shifts relative to the respiratory oscillation φ*_i_* were drawn for each MC from a normal distribution with an SD going from 0.2, in the case of high intensity (this ensured a good locking of the MC population to the respiratory rhythm), to an SD of 5 in the case of low intensity (this induced a broad tuning of MC response relative to the odor onset (as reported in Rospars et al., 2013) and subsequently a broad tuning relative to the respiratory modulation. In the latter case, MCs were not only desynchronized with the respiratory rhythm but also relative to the respiratory rhythm of each other.

##### Centrifugal input modulation

Numerous centrifugal fibers project to the olfactory bulb (Price, 1968; Matsutani and Yamamoto, 2008; Rothermel and Wachowiak, 2014). Although most of the temporally patterned activity in the olfactory bulb is shaped by nasal airflows (Fukunaga et al., 2012; Phillips et al., 2012; Youngstrom and Strowbridge, 2015; Gupta et al., 2015), it has been shown that removing the airflow via a tracheotomy leaves a phase-delayed, respiration-modulated activity in MC spiking discharge (Ravel et al., 1987; Ravel and Pager, 1990) and in the membrane potential of MCs and tuft cells (Phillips et al., 2012), with the latter one being affected by a LOT lesion (Phillips et al., 2012, their Fig. 3). In the model, we introduced this centrifugal modulation through a sinusoidal current targeting only GCs with an average shift of phase compared with the sensory input (ΔΦ; see Results for estimation). It is described as follows in Equation 14:

(Eq.14) $$I_{centrifugal,E,i}(t)=\frac{I_{E,base}+I_{E,\max}}{2}+\frac{I_{E,\max}-I_{E,base}}{2}\left[1+\cos\left(2\pi \cdot f\cdot t+\phi_{i}-\Delta \varphi \right)\right]$$

where *I_E_*_,base_ = −4 nA and *I_E_*_,max_ ranged from −2.5 to 0 nA, depending on centrifugal input strength. These currents are negative to prevent the granule from spiking in response to the sole centrifugal inputs (Pressler et al., 2007) and to account for the very low level of activity of GC under anesthesia (Cazakoff et al., 2014). For each GC, a phase shift, φ*_i_*, was drawn from a random normal distribution with an SD of 0.2 in simulation of anesthetized conditions and an SD of 5 in simulation of awake conditions (Cazakoff et al., 2014). Other connections of centrifugal inputs arriving in the OB on other neuron types were not included in the model, as GC inputs (except those from MC inputs) were thought to be formed mostly by centrifugal inputs in contrast to other modeled neurons (MCs).

##### Model limitations

Several mechanisms can impact the network dynamics and have not been included in the model. Among them we can cite the following: the distribution of local processes along dendrites of MCs and GCs, the contribution of other neuronal populations, and a neuron-specific spatial and temporal description of synaptic inputs received from external structures. Unfortunately, those components have not been well characterized experimentally, and it is thus difficult to include them as necessary components of gamma and beta oscillations presented here. Rather, our aim was to demonstrate that all basic components used in our model are necessary for the network dynamics. The principles of the dynamics can then be drawn and consolidated in order to later allow the addition of supplementary components based on new experimental evidence.

#### Model local field potentials

LFPs issued from network simulations were obtained by convolving MC spike trains with an IPSC-like waveform (same property in time and amplitude as the spike-independent IPSC from GC to MC) and then averaging the obtained waves across the mitral cell population. This method was efficient as it ensured a balanced representation of MC activity at both gamma and beta frequencies in the LFP while also taking into account the time constant of the synaptic inhibition. We used arbitrary units (a.u.) with the same reference scale across all the figures. To preserve both time and frequency information, we used a time–frequency representation that was based upon continuous wavelet transformations. The LFP signal was convolved by a complex Morlet’s wavelet with a time resolution of 5 ms and a frequency resolution of 1 Hz. Using a wavelet ridge extraction, each gamma and beta oscillatory epoch of the LFP was extracted using an energy threshold to detect its beginning and end (Roux et al., 2007). As it was shown to accurately represent the experimental gamma and beta frequency ranges (Neville and Haberly, 2003; Cenier et al., 2009; Martin and Ravel, 2014), the detection boundaries of the model were set to 15-40 and 40-90 Hz, respectively, for the beta and the gamma (which fits well the bimodal distribution observed in the model). This procedure allowed a reasonable estimation of the phasic, temporal, and frequency features of these oscillations. An identical threshold was used for gamma and beta oscillations: it was set to 0.2 a.u. in the absence of slow respiratory modulation and to 0.1 a.u. in the presence of slow respiratory modulation in order to facilitate the detection of transient fast oscillations. Gamma oscillations were detected in the 40-100 Hz frequency range, and beta oscillations were detected in the 15-40 Hz frequency range.

#### Software

Network and neuron equations were implemented under Python version 2.7 using the Brian simulator (Goodman and Brette, 2009). The code for the simulation is available in the model database (https://senselab.med.yale.edu/neurondb/). Euler integration was used with a time step of 0.05 ms.

## Results

MC/GC dendrodendritic interactions were proven to be critically involved in gamma oscillations (Desmaisons et al., 1999; Lagier et al., 2004) and were suggested to be involved in beta oscillations (Fourcaud-Trocmé et al., 2014; Lepousez et al., 2014). Additionally, GCs were shown to display two different modes of dendritic activation (Egger et al., 2003, 2005), as follows: a local mode with a local dendritic depolarization due to MC excitation limited to a single branch of the GC dendritic tree; and a global mode with a full dendritic arbor depolarization caused by GC spikes. In a first step, we hypothesized that when GCs are functioning in the local mode, MCs connected to the same dendritic branch are effectively connected by a weak inhibition that is independent of the GC spike. We showed that this local mode functioning allows the network to oscillate in the gamma frequency range. In a second step, we hypothesized that centrifugal subthreshold excitation of GCs allows them to spike in response to MC excitation and thus to enter the global mode. We showed that this global mode allows the network to oscillate in the beta frequency range. Importantly, while in this study the difference between these two modes relies on the distinct extent of GC dendrite activation, we did not model the details of the GC dendrites. Instead, we implemented the following two distinct types of inhibition: a direct weak inhibition between MCs, accounting for local GC dendrite activation, and a strong inhibition from GC to MC that is activated by GC spikes (Fig. 1*C*,*D*). A sketch of the modeled network and its connections, including these two types of inhibition, is shown in Figure 1 (for details, see Materials and Methods). Finally, as a prominent part of the OB dynamics, we included the respiratory slow rhythm in our model, and studied the influence of the balance between respiratory modulated sensory and centrifugal inputs on the competition between the two fast-oscillatory dynamics.

### Local versus global GC dendritic activation mode can account for the emergence of gamma versus beta oscillation

#### Gamma oscillations can emerge from weak coupling due to local activation of GCs

**Figure 2.**
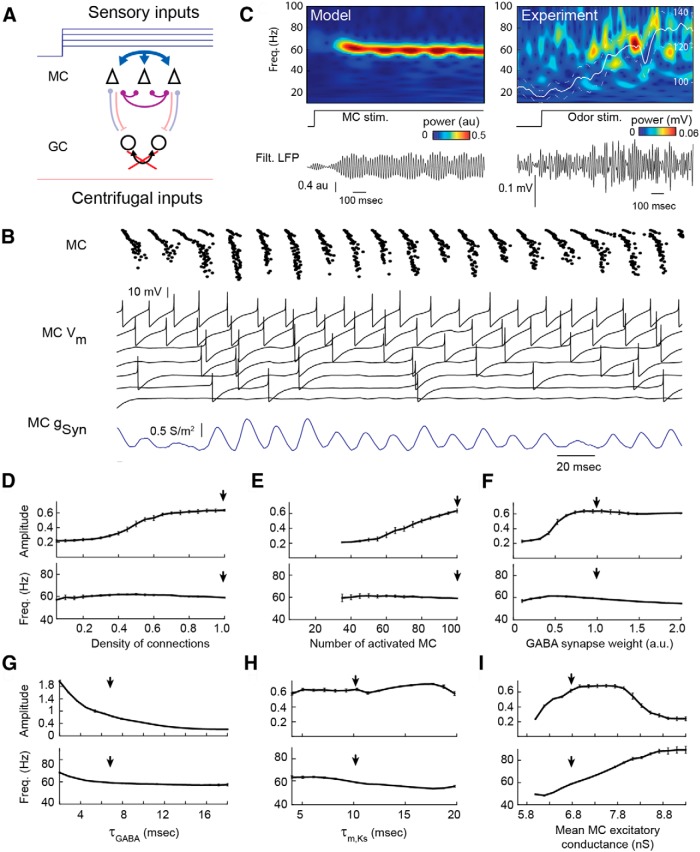
Gamma oscillation emerges from weak coupling between MCs via dendrodendritic synapses. ***A***, Scheme of activated synaptic connections (red arrows) involved in the gamma oscillation. ***B***, Raster images of the 100 MC population (top), examples of MC *V_m_* values (middle), and an example of inhibitory synaptic conductance received by MCs (bottom). ***C***, LFP time–frequency maps show the onset of the oscillation at the gamma frequency (∼60 Hz) following a step (middle trace) of heterogeneous excitatory conductances on MCs in the model (left) or following a constant 2-heptanone-odor (9% of the saturated vapor pressure) stimulation (right) in a tracheotomized rat. White line shows the average time course of gamma maximal amplitude (*n* = 22 recordings from two animals; dotted lines indicate ±SEM) normalized to 100% for amplitudes measured before stimulus onset (time 0). ***D–I***, Effect of the density of connection (***D***), the number of activated MCs (***E***), GABA synapse weight (a.u. are multiples of GABA default conductance; ***F***), the decay time constant of GABA conductance (***G***), the time constant of activation of the slow potassium conductance in MCs (***H***), and the mean MC excitatory conductance (***I***) on the amplitudes (top rows) and frequency (bottom rows) of the detected gamma oscillations. Average values ± SDs are plotted for 30 repetitions of 4 s simulations. The arrow indicates the parameter value used by default from ***D*** to ***I***. Note that when synaptic weights go to 0 (***F***), the gamma amplitude goes to 0.2, which reflects the LFP noise level in the gamma range for constant sensory inputs. Freq., Frequency; stim., stimulated.

In a first set of simulations, the network received only sensory excitation on mitral cells. This mitral input consisted of a range of constant excitatory conductances, which induced an intrinsic firing frequency of MCs ranging between 0 and 70 Hz (for the full characteristics of the MC model, see David et al., 2009; Fig. 1*A*) and bursting patterns, as seen in experimental *in vitro* conditions (Desmaisons et al., 1999; Balu et al., 2004). In such conditions, the synaptic depolarization of GCs via dendrodendritic synapses was not strong enough to elicit GC spikes, and only the direct weak inhibition between mitral cells was brought into play. We modeled this MC–MC interaction using an all-to-all inhibitory coupling (Fig. 2*A*). Random delays of synaptic transmission (drawn uniformly in the 5-13 ms range, independently for each MC pair) were used to reflect the actual indirect and asynchronous nature of inhibition from GCs to MCs. The weak MC coupling, along with the heterogeneous input excitatory conductances received by MCs, contributed to the heterogeneous characteristic observed in MC activities (for MC *V_m_* examples, see Fig. 2*B*). When considering LFP signal, a gamma oscillation at ∼60 Hz in frequency emerged after the onset and was maintained during the stimulation (Fig. 2*C*, left). This oscillation is due to an autoentrainment mechanism. Precisely, because inhibition is weak, individual MC spike patterns are only slightly modulated by the inhibitory interactions. Each MC thus generates an approximate oscillatory input that tends to entrain other MCs spiking at a close firing frequency rate. In turn, entrained MCs reinforce the global oscillation and favor the entrainment of additional MCs. Interestingly, a previous study of this MC model (David et al., 2009) showed that the efficacy of such an entrainment depends on an entrained MC firing rate and oscillation frequency. Indeed, due to MC resonant properties, it reaches its maximal strength when the oscillation frequency is at ∼60 Hz. This explains the large oscillation at ∼60 Hz observed in Fig. 2*C*. Only a subset of cells was perfectly entrained and discharged once every cycle or every two cycles. Because most the cells cannot perfectly follow the LFP oscillation (because of the difference between their intrinsic firing rate and the LFP oscillation frequency), this leads to a more irregular discharge with skipped cycles or two spikes in a cycle (for examples, see Fig. 2*B*). This is in agreement with the results of a previous study (David et al., 2009) on MC spike phase locking during gamma oscillations *in vivo* in freely breathing anesthetized rats.

We next wondered whether we could experimentally reproduce this network model behavior in urethane-anesthetized animals, where GC excitability is known to be highly reduced (Kato et al., 2012; Cazakoff et al., 2014). We performed a tracheotomy and applied a continuous odorant stimulation (using a constant aspiration) to produce a prolonged excitation on MCs, which was also independent from the respiratory rhythm. A deafferentation of the OB was also performed by lesioning the peduncle to insulate the OB from centrifugal inputs. We observed, as predicted by our model, that in such conditions, odorant stimulation induced a continuous gamma oscillation (Fig. 2*C*, right, *n* = 2 animals, *n* = 22 stimulations; for comparison with data published elsewhere, see Table 1).

**Table 1:** Summary of findings and associated experimental evidences

Model outputs	Experimental data from the group	Figure	Other comparable reports
Generation of sustained gamma rhythm by continuous odorant stimulation	Present data (*n* = 2)	2C	Neville and Haberly, 2003
Generation of beta oscillations by increased GC excitability	No direct evidence	3D	Only indirect evidences: Martin et al., 2006, Kay and Beshel, 2010
Change of GC excitability over a respiratory cycle	Present data (*n* = 18)	4C	Only indirect evidence:[Bibr B23][Bibr B105]
Gamma–beta sequence locking on respiration under anesthesia	Buonviso et al., 2003 (*n* = 33) Cenier et al., 2008 (*n* = 14)	4E,F	
Correlation between gamma/beta power and odor intensity	Courtiol et al., 2011a(*n* = 12)	5A,C	Neville and Haberly, 2003
Strong decrease of beta oscillations after disruption of OB centrifugal inputs	Present data (*n* = 6)	6A,B	[Bibr B86][Bibr B76]

*n*, number of animals.

To better understand the network parameters used in our model that were important to precisely control gamma oscillation properties, we made simulations systematically varying the network parameters one by one. As described above, the mechanism leading to gamma oscillation is an autoentrainment mechanism; thus, LFP frequency should depend mainly on MC entrainment properties (David et al., 2009) and MC firing rates. Because the weak inhibition tends to modulate spike patterns with only a minor impact on the mean firing rates (David et al., 2009), the oscillation frequency was barely affected by synaptic parameters (density of connection; Fig. 2*D*), weak inhibition amplitude (Fig. 2*F*), inhibition decay time constant (Fig. 2*G*), or the number of activated MCs (Fig. 2*E*). Regarding MC entrainment properties, increasing the time constant of the slow potassium current activation, which is responsible for MC resonant properties, was previously shown to shift the maximal efficacy of MC entrainment to lower frequencies (David et al., 2009). This parallels the small decrease of LFP frequency found in our network for the larger time constant of the slow potassium current (Fig. 2*H*). Finally, the parameter with the most influence on LFP frequency was the strength of mean MC excitatory conductance (Fig. 2*I*), which was similar to what has been reported in other OB models (Börgers and Kopell, 2003; Brea et al., 2009; Fourcaud-Trocmé et al., 2011). Indeed, varying the MC input conductance strongly affected the MC firing rate. At low-input excitatory conductance, MCs tend to discharge by small bursts with an intraburst frequency of ∼40 Hz (Fig. 1*A*), which explains both why the LFP oscillation frequency did not decrease below 40 Hz and why some bursts of gamma oscillation occur at a much slower frequency (∼8 Hz). At high-input excitatory conductance, faster MCs tried to entrain the whole network at their intrinsic firing rate, and the resultant LFP frequency was intermediate between the highest MC firing rate and the best entrained frequency (60 Hz, defined by MC intrinsic properties). However, we must emphasize that at higher LFP frequencies there was a strong decrease of LFP amplitude (Fig. 2*I*). This was due both to the less effective MC entrainment at frequencies higher than 60 Hz, and to the weak inhibition delay and rise times, which began to last longer than one LFP cycle. Thus, high-frequency oscillations were strongly attenuated and tended to disappear in LFP noise.

Overall, we showed that weak coupling between MCs led, through an autoentrainment mechanism, to the emergence of network oscillations, specifically in the gamma frequency range.

#### Beta oscillations require cortical feedback and full GC activation

**Figure 3. F3:**
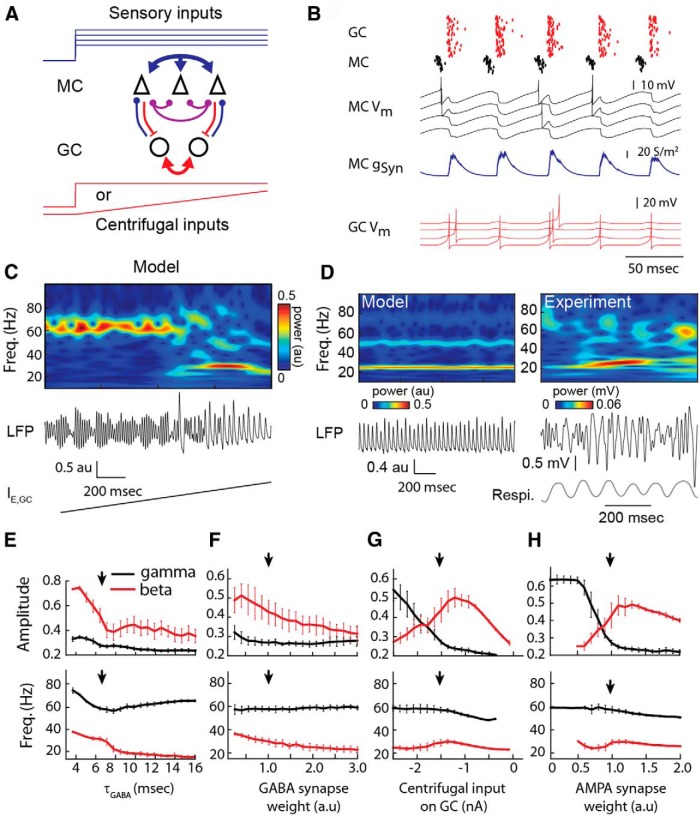
Beta oscillations compete with gamma oscillations when GCs start spiking. ***A***, Scheme of activated synaptic connections (red arrows) involved in the beta oscillations. The centrifugal inputs on GC are added compared with Figure 2. ***B***, Raster of the MC (black dots) and GC (red dots) populations, *V_m_* of four MCs, inhibitory synaptic conductances received by MCs, and *V_m_* of four GCs. ***C***, Wavelet transform and LFP show the response of the network to a ramp of excitatory current uniformly imposed onto the GC population (*I_E_*_,GC_). ***D***, Wavelet transform and LFP during stable beta oscillations in the model (left) and in awake behaving rats after ethyl-benzoate odor sampling associated to reward (right) as an illustrative example of common beta induction in the OB. Note that the oscillation covers multiple respiratory cycles (Respi. signal). ***E–H***, Effects of the decay time (***E***) and weight (***F***) of GABA spike-dependent IPSCs from GC to MC, the amplitude of the centrifugal inputs on GC (***G***), and the amplitude of AMPA EPSCs from MC to GC (***H***) on amplitude (top rows) and frequency (bottom rows) of detected oscillations in the gamma (black trace) and beta (red traces) frequency ranges. The arrow indicates the parameter value used by default from ***E*** to ***H***. Average values ±SDs are plotted for 30 repetitions of 4 s simulations. Freq., Frequency.

The clear distinction of frequency bands covered by gamma and beta oscillations *in vivo* suggests the existence of two completely different generation mechanisms that are supported by distinct subnetworks in the mitral–granule network (Fourcaud-Trocmé et al., 2014). However, no mechanism has convincingly explained this clear-cut separation. We tested whether the spiking of GCs underlies the emergence of the beta oscillations as has recently been hypothesized (Lepousez et al., 2014). Compared with the previous network configuration, we added a centrifugal contribution as an excitatory input mimicking the barrage of EPSPs from centrifugal fibers to GCs (Figs. 1*B*, 3*A*, upward red arrow; for details, see Materials and Methods). Although these inputs are phase modulated by the respiratory cycle (Rothermel and Wachowiak, 2014), they were first modeled as a homogeneous steady excitatory conductance on GCs (Fig. 3*A*). In such conditions, GCs were able to fire action potentials (Fig. 3*B*) when their total excitation (centrifugal excitation plus MC excitation) was large enough. In turn, this GC spiking elicited strong inhibition of MCs.

In order to assess the effects of the switch of GCs from a nonspiking to a spiking regime, we added a ramp of excitatory current impinging on GCs, which brought them closer to their spiking threshold (Fig. 3*C*). In this novel network state, MC spikes induced GC spikes, which in turn inhibited MCs sufficiently to transiently block their discharge. A new cycle could start when GC inhibition sufficiently decayed (Fig. 3*B*). This type of oscillation has most of the properties of a PING mechanism (Börgers and Kopell, 2003). As a consequence, network dynamics were driven by the interplay between MC excitation and spike-induced GC inhibition, while gamma oscillations were disrupted by the now powerful inhibitory input received from GCs. This resulted in a sudden shift in the network oscillation from the gamma regime previously observed to a beta regime (15-40 Hz), as illustrated in Figure 3*C*. Using a constant and strong centrifugal input, the network displayed a beta oscillation of stable amplitude and frequency (Fig. 3*D*, left).

*In vivo* experimental conditions promoting a strong centrifugal input on GCs are the moments when an animal samples an odorant while it is awake compared with anesthetized (Lowry and Kay, 2007; Cazakoff et al., 2014) or while the odor circuit is reinforced by learning (Cauthron and Stripling, 2014; Lepousez et al., 2014). Comparing our model with actual data, we noted that in such conditions we could also get a long beta oscillation in the bulbar network LFP (Fig. 3*D*, right; for comparison with data published elsewhere, see Table 1).

Finally, we made systematic simulations of the model by varying different parameters one by one in order to study both beta oscillation properties and the competition between gamma and beta oscillations. Figure 3*E–H* displays a summary of these simulations. We observed that the beta oscillation frequency depended critically on both the time constant of inhibition (Fig. 3*E*) and the weight of the GABA inhibition (Fig. 3*F*), but less (the nonmonotonic effect) on the weight of excitatory centrifugal inputs (Fig. 3*G*) or an AMPA excitatory MC–GC synapse (Fig. 3*H*). However, we observed that the balance between the amplitudes of beta versus gamma oscillations was tightly controlled by parameters that control the GC firing (i.e., the centrifugal input strength on GCs; Fig. 3*G*) and the AMPA conductance (Fig. 3*H*). Indeed, the combination of both excitatory inputs on GCs has to be large enough to elicit GC spiking and thus makes beta oscillations emerge. We intentionally did not include any random process on the temporal course of the peripheral or centrifugal inputs in order to better isolate the phenomenon described from sources of variability (Figs. 2, 3). This yields relatively narrow bands of activity in frequency and amplitude, and explains some of the dissimilarities with the presented experiments on top of an overall good concordance.

Overall, we showed that increased excitation on GCs could lead to a GC spiking regime that made the network oscillate in the beta frequency range. Beta oscillation properties were mainly determined by intrinsic network synaptic properties, while the balance between beta and gamma oscillations was tightly controlled by both network peripheral and centrifugal excitatory levels.

### The phase shift between sensory and centrifugal inputs explains the gamma–beta sequence over a respiratory cycle

**Figure 4. F4:**
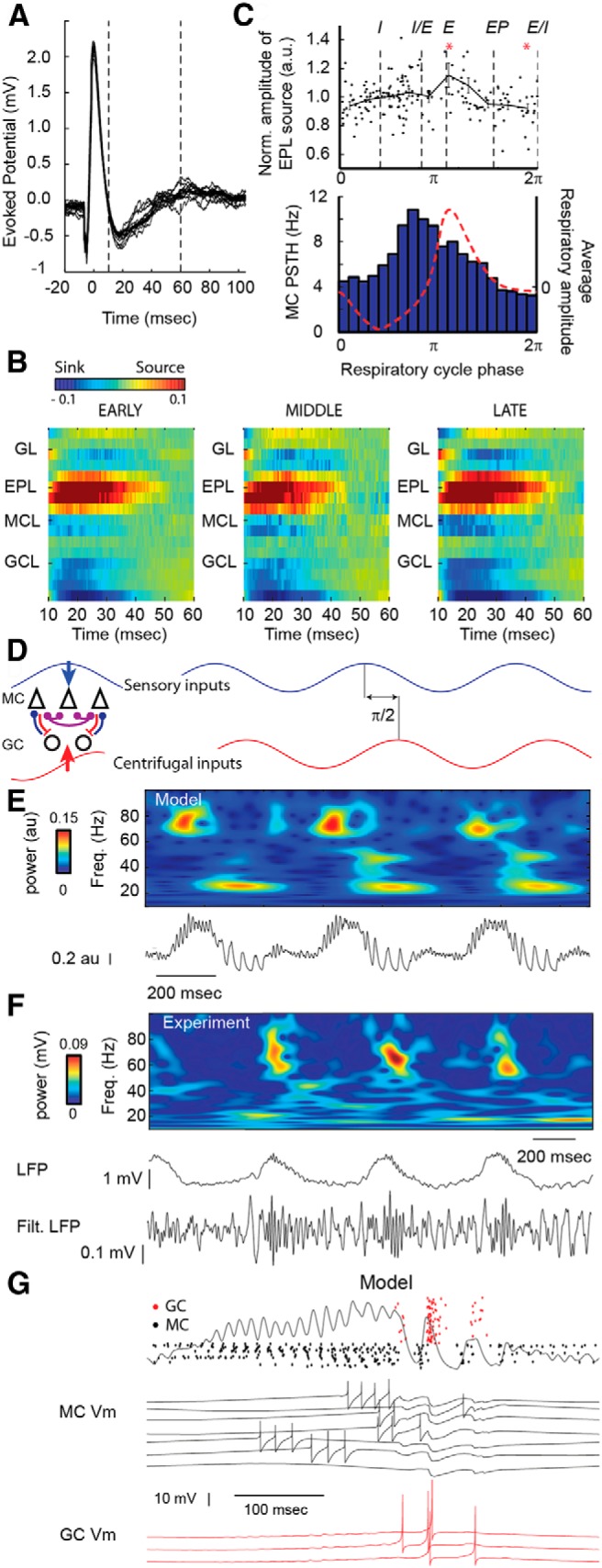
Oscillation sequence during the time window of a respiratory cycle reproduced in the model. ***A***, Examples of recordings in the OB GCL of a freely breathing urethane-anesthetized rat during LOT electrical stimulations at different respiratory phases (*n* = 14 traces are superimposed). Time 0 is aligned for each trace on the time of the positive peak. Dashed vertical lines indicate the time limits considered for the CSD around the negative peak. ***B***, Current source density maps across olfactory bulb layers on the 10–60 ms interval poststimulation for three distinct respiratory phases (early, 0–0.5), middle (0.5–0.7), and late (0.7–1), respectively. Maps are averages of LOT stimulations across different recording sessions (*n* = 18) across layers, as follows: GL, EPL, MCL, and GCL. Current sink and source amplitudes are color coded (color bar). ***C***, Top, EPL current source amplitude of the negative evoked potential as a function of the respiratory phase of LOT stimulation. Vertical dashed lines indicate the I maximum, the I/E transition, the E maximum, the EP, and the E/I. Red asterisks indicate significantly different bins (Wilcoxon test, *p* < 0.01). Bottom, MC discharge during the respiratory cycle. The dashed red line is the average respiratory amplitude (inspiration is downward). ***D***, Scheme of the sensory and cortical inputs as two sinusoids here at 2 Hz shifted by π/2. ***E***, Gamma and beta sequence through three consecutive simulated respiratory cycles. The jitter of phases of MC is 2.5 (see Materials and Methods). ***F***, Sequence of gamma and beta oscillations measured experimentally *in vivo* in response to heptanal odor stimulation (9% of SVP) and corresponding raw LFP, and filtered (10–90 Hz) LFP over approximately three respiratory cycles. Note the locking of gamma and beta to particular phases of the respiratory modulation. ***G***, Raster images of GC firing (red) and MC firing (black); LFP trace is superimposed. Black, Seven examples of MC *V_m_*; red, three examples of GC *V_m_*. The time period corresponds to the one indicated by the gray bar in ***E***.

Having described gamma and beta oscillations in our model, we then wanted to understand their relationships with the slow respiratory rhythm. Our first objective was thus to assess how the influence on this slow rhythm in our network should be modeled. *In vivo* experiments have extensively shown a respiratory slow modulation of sensory input (Carey et al., 2009; Courtiol et al., 2011a; Briffaud et al., 2012; Phillips et al., 2012; Rojas-Líbano et al., 2014). Besides, although a centrifugal modulation of the OB at the respiratory rhythm is debated (Ravel et al., 1987; Sobel and Tank, 1993; Phillips et al., 2012), a respiration-locked modulation of GCs by centrifugal inputs probably exists (Rothermel and Wachowiak, 2014). Moreover, some data suggest that this centrifugal respiratory modulation could be phase shifted relative to the respiratory modulation of sensory input under anesthesia (Ravel and Pager, 1990; Phillips et al., 2012). In order to investigate to what extent respiratory modulation could be phase shifted between sensory and centrifugal inputs, we proposed to measure experimentally, in freely breathing anesthetized rats, how GC excitability changed along the respiratory cycle. We reasoned that if GC excitability was not constant but rhythmic during the whole respiratory cycle, the late synaptic current response to LOT electrical stimulation (corresponding to GC activity) should be larger during high GC excitability phases. We thus recorded OB LFP in response to LOT stimulation at distinct respiratory phases (for examples of recordings, see Fig. 4*A*) with a 16-channel silicon probe. We performed a CSD analysis of the late (10-60 ms) part of the LOT-evoked responses grouped according to their respiratory phases (see Materials and Methods). The amplitude of this late component in the external plexiform layer was previously interpreted either as the inhibitory input on the MC dendrites (Nicoll, 1972) or as a depolarization following centrifugal excitation of the GCs (Nakashima et al., 1978; Aroniadou-Anderjaska et al., 1999; Uva et al., 2006). Because these two origins could not be disentangled in our data, we considered that they both contributed to generating the main negative component of the LOT-evoked response.

The resultant CSD maps (Fig. 4*B*) displayed a strong current source in the EPL resulting from a mix of the opening of GC–MC inhibitory synapses and of granule cell centrifugal excitation (for details, see Materials and Methods). Interestingly, the overall CSD pattern did not change across respiratory phases except for its amplitude, indicating that evoked recurrent inhibition and GC-evoked centrifugal excitation were not constant at each respiratory phase. To quantify this change, we measured the amplitude of the current source in the EPL as a function of the respiratory phase. We observed a significant (mean ± SEM, 22 ± 11%) modulation of the EPL current source amplitude (Fig. 4*C*, top; *n* = 180 LOT stimulations; Kruskal–Wallis test, *p* < 0.01), which peaked at the initial part (1.1 π) of the expiration phase and dipped at the end of expiration (E/I; 0 π, unpaired Wilcoxon test, *p* < 0.01; *n*_0π_ = 18, *n*_1.1π_ = 10). In contrast, we did not find any significant changes in the amplitude of the EPL current sink around the peak of the LOT-evoked response (from −2 to 10 ms; data not shown; *n* = 180 LOT stimulations; Kruskal–Wallis test, *p* = 0.90), which reflects the amount of evoked excitatory current from MC to GC (Nakashima et al., 1978). We thus concluded that the slow modulation of the LOT-evoked EPL source amplitude that we observed was due mainly to a slow modulation of centrifugal inputs onto GCs (either by increasing their excitability, and thus promoting recurrent inhibition, or by modulating their sensitivity to centrifugal feedback). When comparing these data with the firing rate of MCs along the respiratory cycle, we observed that the MC firing rate was maximal at the I/E transition (0.74 π; Fig. 4*C*, bottom). Finally, based on these measurements, we concluded that excitatory slow modulation of MCs and GCs, sensory and centrifugal, respectively, could be considered as shifted by approximately a quarter of the respiratory cycle phase. These findings were also corroborated by data from another study on MC and GC unit activity (Ravel et al., 1987), which proved the existence of a modulation of bulbar activity independent of sensory inputs and phase delayed relative to the I/E transition in the respiratory cycle.

To introduce these phase-shifted modulations in the last network configuration, we replaced (1) the constant sensory conductance by the sensory respiratory modulation as a sine wave modulation of excitatory inputs onto MCs and (2) the constant centrifugal conductance by a centrifugal periodic modulation of excitatory inputs onto GCs phase shifted by −π/2 relative to sensory input (for details, see Materials and Methods; Fig. 4*D*). Note that this centrifugal modulation remained at a subthreshold level (i.e., did not evoke spikes) for GCs. This allowed us (Fig. 4*E*) to reproduce in the model the sequence of fast oscillations usually observed experimentally at each respiratory cycle in response to odor stimulation in anesthetized rats (Fig. 4*F*). The bursts of gamma oscillations were phase locked to the simulated I/E transition, and beta oscillations were locked to the simulated E value. As described in Figures 1 and 2, MCs tended to be more active during gamma oscillations and less active during beta oscillations (Fig. 4*G*). GCs had an opposite pattern with a peak of activity during the beta oscillations. At the unit level, the model also matched the following experimental observations: (1) the majority of MCs fired at the I/E transition were locked in phase to the gamma oscillations; and (2) MCs fired during the expiration phase were locked to the beta oscillations.

### The strong impacts of odor intensity, odor valence, and sniffing strategy on the dynamic state of the network are explained by the balance between sensory and cortical inputs

Simulations from the previous section showed that our model could accurately capture the alternation of gamma and beta oscillations shaped by the respiratory slow modulation in a standard anesthetized animal preparation. In the awake preparation, such alternations do not appear so regularly, but switches between both regimes are well described and gamma bursts are also locked to the respiratory rhythm (Rojas-Líbano and Kay, 2008; Martin and Ravel, 2014). To gain insight into the underlying mechanisms of the competition between beta and gamma oscillations in awake animals in a more functional context, we explored the model dynamics when the animal is facing changes in network input parameters similar to those that would occur in different experimental and behavioral conditions. In particular, we were interested in (1) changes in sensory input intensity, which can be due to changes in odor concentration or/and nasal flow rate; (2) changes in centrifugal inputs, which can be affected by learning, contextual association, or pharmacological manipulations; and finally (3) changes in sniffing frequency, as happen when animals are actively exploring an odor source.

#### Changes in the strength of afferent inputs

**Figure 5. F5:**
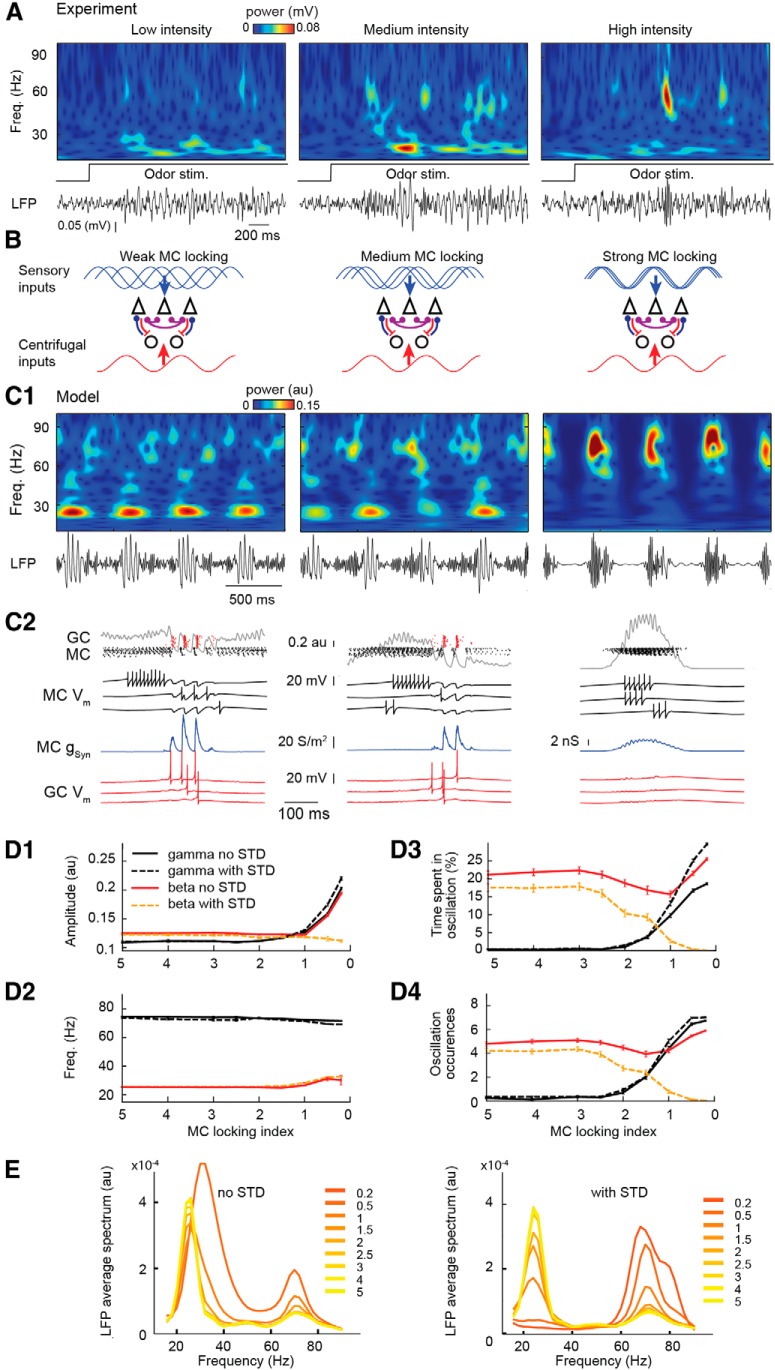
Degree of MC locking to the respiration reproduces the odor stimulation intensity on network oscillations. ***A***, In tracheotomized urethane-anesthetized rats, the variation of odor intensity (odorant used: isoamyl-acetate), using different flow rates, controls the patterns of gamma and beta oscillations. Low intensity more likely induces beta oscillations, whereas higher intensity more likely gradually induces gamma oscillations (middle and right). ***B***, Scheme of modulation of sensory and cortical inputs. Increasing odor intensity is simulated by gradually increasing the level of locking (jitter of phase) to the respiratory cycle of MCs (first row), whereas the cortical afferent input is maintained constant (second row). ***C1***, LFPs and their wavelet transforms, for the following three simulated conditions: from low (left, jitter of phase = 5), intermediate (middle, jitter of phase = 1.5) to high (right, jitter of phase = 0.5) levels of MC locking progressively favors gamma compared to beta oscillations. ***C2***, Corresponding raster images and trace examples are represented below each case. ***D***, The effect of MC locking to respiration on amplitude (***D1***), frequency (***D2***), time spent in oscillation (***D3***), and the number of occurrences of gamma (black) and beta (red) oscillations (***D4***). Data are plotted as the average ± SEM on 100 simulations of 3 s each after 1 s of stabilization. Introducing STD (dashed lines; gamma in black and beta in yellow) at the AMPA synapse from MC to GC reduces the chances of beta oscillation for high locking of MCs to the respiratory cycle. ***E***, Power spectra for the full range of MCs locking to the respiratory cycle (see color code) without STD (left) and with STD (right). stim., Stimulation.

Experimentally, the strength of afferent input can be varied by changing nasal flow rate (Fig. 5*A*; Courtiol et al., 2011a) or/and odor concentration (Neville and Haberly, 2003). In both cases, it has been reported that the stronger the afferent input (high flow rate or high concentration), the stronger the gamma oscillations appear. Alternatively, the weaker the afferent input (i.e., low flow rate or low concentration), the stronger beta oscillations appear (Neville and Haberly, 2003; Courtiol et al., 2011a). In order to explain such a competing mechanism able to control the predominance of gamma or beta oscillations, we simulated a change in input intensity (i.e., either odor concentration or nasal airflow) by increasing the strength of phase locking to the respiration cycle (see Materials and Methods) of the sensory stimulations across MC population, while keeping unchanged the temporal course of centrifugal inputs for the different afferent input intensities (Fig. 5*B*).

For weak afferent inputs, the MC inputs poorly locked to each other failed to induce detectable gamma oscillations, whereas beta oscillations were robustly present at each respiratory cycle (Fig. 5*C1*, left). When the strength of the afferent inputs increased and the locking of MCs to the respiration cycle increased, gamma oscillations appeared and increased in amplitude (Fig. 5*C1*, middle and right, *D*). This tendency for the gamma oscillations corresponded well with the general tendency observed in experimental conditions (Courtiol et al., 2011a), where a higher proportion of MCs (81%) respiration-locked for high flow rates compared with lower flow rates (61%). For beta oscillations, the results are less simple. In fact, when the strength of the afferent inputs increased and the locking of MCs to the respiration cycle increase, the number of occurrences of beta waves initially tended to decrease, but this proportion rose again for very high locking levels (Fig. 5*D4*, red lines). This result is in apparent contradiction with some experimental observations in anesthetized rats (Neville and Haberly, 2003; Courtiol et al., 2011a) showing that high odor concentration [in a moderate (i.e. not at saturated vapor pressure) range of concentration] mainly diminishes the number of beta occurrences (see opposite tendency for odors at saturated vapor pressures in Lowry and Kay, 2007). This discrepancy between the model and experimental results could be explained by the fact that although the locking of MCs to the respiratory cycle increased and generated high-amplitude gamma oscillations, synchrony among the cells in the MC population through the gamma cycle could induce highly synchronous inputs to GCs, which are strong enough to elicit occasional GC firing (not shown). This yielded occurrences of beta oscillations concomitant with gamma oscillations also observed in the power spectra (Fig. 5*E*, left). To overcome this discrepancy, we searched for a mechanism that was able to decrease the total excitation of GCs while gamma oscillation was strong. Consistent with experiments that reported a short-term depression (STD) on the MC–GC AMPA synapse (Balu et al., 2007), we added this mechanism to the model (see Materials and Methods). The main STD effect was to strongly decrease the amplitude of the AMPA EPSPs on GCs when presynaptic spike intervals from MC to GC reached values as fast as the gamma range frequencies. As expected from results seen in Figure 3*H*, this decrease in MC–GC excitation induced by the STD resulted in a drastic decrease in the occurrence and power of beta oscillations when the locking of MCs to the respiratory cycle increased ([Fig F3][Fig F4][Fig F5] yellow lines, *E*, right) compared with conditions without STD ([Fig F3][Fig F4][Fig F5]red lines, *E*, left). Consequently, STD at the level of the MC–GC synapses appears as an additional efficient mechanism to regulate the competition between beta and gamma oscillations.

#### Changes in the amplitude of centrifugal afferent inputs

**Figure 6.**
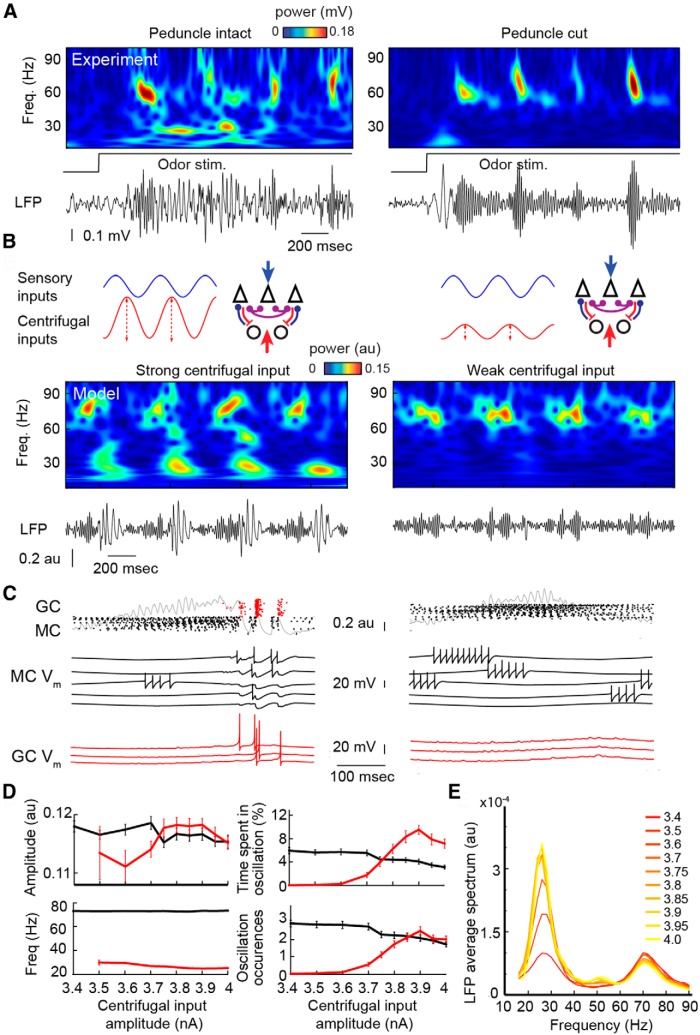
Effects of centrifugal inputs on GCs during the respiratory cycle. ***A***, Experimental LFP and wavelet transforms for the same odor stimulation (2-heptanone in the OB while the peduncle is intact (left) or cut (right; *n* = 6 rats). For more details, see Table 1. ***B***, Up, Scheme of the modulation received by MC and GC for strong (left) or weak (right) centrifugal inputs in the model. Down, Wavelet transforms and LFPs for strong (3.9 nA; left) and weak (3.5 nA; right) centrifugal afferent inputs on GCs. The jitter of phase is 1.5. The centrifugal input amplitude (dashed lines) is given relative to the basal level of centrifugal input sine wave (chosen at ∼4 nA). ***C***, Examples of raster images and *V_m_* traces for both MCs (black) and GCs (red) for strong (left) and weak (right) cortical inputs. LFP traces are superimposed on rasters. ***D***, Effect of cortical input [maximum current (in nA) relative to the minimum level] on oscillation amplitude, frequency, time spent in oscillation, and number of gamma (black) and beta (red) oscillation occurrences. Data are plotted as the average ± SEM on 100 simulations of 4 s each. ***E***, Global power spectra of the LFP from low-amplitude (red) to high-amplitude (yellow) cortical inputs. Freq., Frequency; stim., stimulation.

Odors with high contextual meaning (strong valence) either acquired or innate (which induce fear, for example) are able to evoke a drastic change in dynamics from gamma to beta oscillations (Zibrowski and Vanderwolf, 1997; Ravel et al., 2003). This behavioral and dynamic conditioning was found to be highly dependent on centrifugal fibers from the piriform cortex in awake conditions (Martin et al., 2006) as well as in anesthetized conditions (Neville and Haberly, 2003). As an example, in experimental conditions when centrifugal afferents from the piriform cortex to the OB are intact, beta oscillations could emerge under urethane anesthesia in response to some specific odors (Fig. 6*A*, left, example). The lesion of these centrifugal afferent fibers constrains the system to the sole expression of gamma oscillations for the very same odorant stimulation (Fig. 6*A*, right), an effect that has been well described by Neville and Haberly (2003). In our model, we assessed the effect of a change in the strength of centrifugal inputs by varying the amplitude of the excitatory conductance received by GCs (Fig. 6*B*, top panels), while keeping the dispersion of phases of the sensory modulation on MCs at an intermediate level. While centrifugal inputs were increased (Fig. 6*B*,*C*, compare left, right), the beta oscillations were enhanced and the gamma oscillations were decreased. Systematically varying the amplitude of the inputs of centrifugal afferents (Fig. 6*D*,*E*) indicated that the total time spent in beta oscillations increased from 0 to 9.4 ± 0.9% of the total simulation time and the total time spent in gamma oscillations decreased, from 6.0 ± 0.5% to 3.2 ± 0.4% compared with conditions where the centrifugal afferents were absent or not sufficient to induce GC spiking (Fig. 6*C*, compare right, left). The amplitude and frequency of the individual gamma and beta oscillations were only slightly affected by the increase of centrifugal feedback (Fig. 6*D*, left). This provided evidence that the mechanisms underlying the formation of beta oscillations can critically depend on both centrifugal afferents and the intrinsic dynamics of the olfactory bulb.

#### Changes in the frequency of sensory and cortical inputs

**Figure 7.**
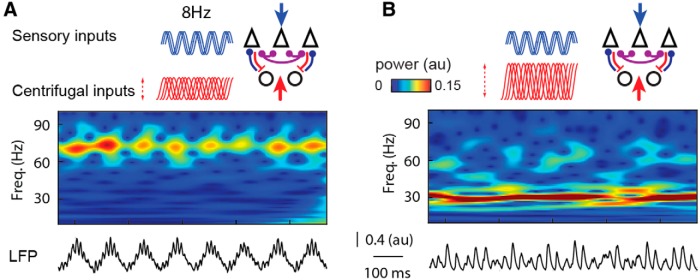
Effect of centrifugal inputs in awake-like sniffing conditions. ***A***, Simulation for a sensory modulation frequency of 8 Hz with cortical afferent inputs on GCs desynchronized relative the respiratory cycle (jitter of phase: for MCs = 1.5; for GCs = 5). This condition generates mainly gamma oscillations when cortical afferent inputs are low. ***B***, Same model generates relatively continuous beta oscillations when centrifugal afferent inputs are increased. Here, maximal sensory input conductances *g*_input,_*_E_*_,max_ ranged from 6.1 to 7.6 S/m^2^. Freq., Frequency.

Awake behaving conditions are associated with different sniffing strategies and in particular with changes in the sniffing frequency (Youngentob et al., 1987; Courtiol et al., 2011a, 2011b). Therefore, we wanted to know whether our model of gamma and beta competition mostly compared to well described anesthetized conditions could also be robust in brain state changes and applied to awake conditions because the goal of the model is to extend its scope to the functional context. Those are characterized by a faster respiratory modulation (Rojas-Líbano and Kay, 2012; Rojas-Líbano et al., 2014), a broader tuning of GC activity relative to the respiratory cycle, and an increase of GC activity (Cazakoff et al., 2014). Here the respiratory modulation was set at 8 Hz and the relative timing of individual cortical excitatory inputs on the GC was desynchronized relative to the respiratory cycle (see Materials and Methods). In conditions of weak or absent centrifugal afferents, we observed that discontinuous oscillations in the gamma frequency range dominated the network activity (Fig. 7*A*). The gamma bursts were still locked to the respiratory modulation in a way that was similar to the locking of gamma bursts to the theta rhythm observed in awake conditions (Lepousez and Lledo, 2013; Manabe and Mori, 2013). When the level of centrifugal excitatory input on GCs was enhanced to favor GC spiking, the network activity switched to relatively continuous beta oscillation overlapping several respiratory cycles (Fig. 7*B*). This captures well the experimental observation in awake animals where the beta oscillations expand over several respiratory cycles (Fig. 3*D*, right; Martin et al., 2004, 2006).

**Figure 8.**
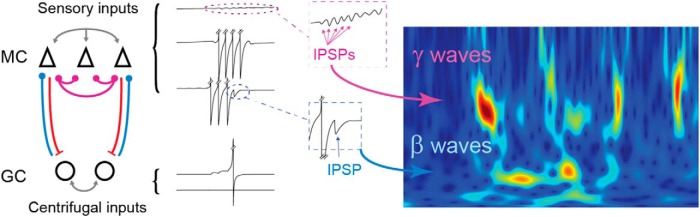
Multimodal inhibition (blue or purple) in the olfactory bulb between granule (GC, circles) and mitral (MC, triangles) cells generates two types of inhibition (IPSPs) that can induce gamma or beta waves in response to odor stimulation.

Overall, the proposed mechanisms unraveled by anesthesia can sustain the dynamics associated with more complex physiological contexts, including a higher frequency of sniffing or broader GC tuning on respiration observed in behaving animals responding to an odor.

## Discussion

In this study, we used a simple but realistic model of the OB to study the emergence and competition of two fast oscillatory processes identified as gamma and beta oscillations. Our model captured these two essential dynamic features relative to odor processing through the window of a respiratory cycle. The parsimony and flexibility of the model, while accurately accounting for the main aspects of OB dynamics, make it an important step in the way of reducing neuronal networks to their essential computational properties. The main model hypothesis is that both oscillations depend on the MC–GC interactions but with a GC regime either spiking or nonspiking. In the nonspiking regime, weak synaptic coupling inhibition allows the emergence of gamma oscillations with characteristics of an autoentrainment process. In contrast, in the GC spiking regime, MCs sufficiently excite the GCs such that the latter discharge and induce a strong inhibitory input that silences the MC population and generate beta oscillations (Fig. 8). We then enter a PING regime (Börgers and Kopell, 2003), where both populations discharge alternatively. We showed that the dynamics of both types of oscillations, gamma and beta, were remarkably stable as a function of most of the network parameters tested. However, their occurrence depended strongly on the network peripheral sensory inputs and centrifugal inputs. In particular, gamma oscillations required a sufficient activation of MCs, while beta oscillations required a sufficient activation of GCs (Figs. 2, 3). Based on novel experimental data showing a phase shift between MC and GC external stimulation (Fig. 4), we showed that the model could account for the alternation of gamma and beta oscillations during a respiratory cycle as observed *in vivo*. Finally, our model accurately captures the competition between gamma and beta oscillations when sensory or centrifugal inputs are modulated, such as in different natural conditions involving odor features and behavior (Figs. 5–7). Overall, this model very closely approaches OB dynamics observed *in vivo*, and can thus be used to interpret present and future experiments.

### Model construction: a trade-off between complexity and necessity

The model used in the present study makes a number of assumptions regarding the underlying biophysical mechanisms that need to be discussed. First, the weak inhibition independent of GC spikes and a stronger inhibition dependent on GC spikes were dissociated. This distinction has been made based on *in vitro* calcium imaging experiments (Egger et al., 2003, 2005) that showed that GC dendrites can have a local activation with local depolarization spreading only to spatially close spines on the dendrite, or a more global mode where the full GC dendritic arbor is activated by a GC spike. In real GCs, both mechanisms activate overlapping sets of synapses and are thus not additive at the MC soma level, as in our model. However, we observed that beta dynamics were generally overwhelming gamma dynamics that are likely to be similar to the experimental dynamics because broad dendritic activation should overwhelm local dendritic activation. A more realistic description of this competition could be made with a detailed model of GCs, but this would require the fine tuning of many parameters that are not well known experimentally (in particular, regarding granule dendrite internal dynamics). An alternative solution proposed in the literature is the use of graded synapses for GC–MC inhibition (David et al., 2008; Brea et al., 2009; Fourcaud-Trocmé et al., 2011), to which our inhibitory synapses can be compared. Our weak inhibition corresponds to a weakly activated graded synapse, whereas our spike-dependent inhibition corresponds to a saturated graded synapse. And indeed, these two modes relate, respectively, to the gamma and beta regimes described in the study by Fourcaud-Trocmé et al. (2011). Finally, recent experimental evidence points toward the activation of distinct but overlapping sets of cells or synapses during gamma and beta oscillations in the OB (Cenier et al., 2009; Fourcaud-Trocmé et al., 2014), which supports the distinction of both mechanisms. Overall, despite its simplicity, the distinction between a weak and a strong inhibition mechanism accurately captures the interaction between gamma and beta oscillatory regimes in various experimental conditions. Additional mechanisms, such as the GCs in NMDA receptors, facilitate centrifugal inputs (Balu et al., 2007), and their interactions may also be included for future and more comprehensive studies of the processes regulating OB dynamics.

A second point, which deserves full attention, is the exclusion from the model of other neuron types to explain the generation of the oscillatory rhythms, as follows: interneurons other than GCs and TCs in particular; and the recent characterization of some of the interneurons in the physiological context of the olfactory bulb (Kato et al., 2013; Miyamichi et al., 2013). Similar to GCs, parvalbumin (PV)-positive interneurons have been described as being responsive to odors and connecting principal cells in the external plexiform layer. Their involvement in the generation of IPSCs on MCs and on gamma oscillations (Lagier et al., 2004) could be central if they play a key role, like PV-positive cells of the neocortex, in gamma generation (Cardin et al., 2009). Similarly, it is likely that other neuron types in the glomerular layer, like short-axon cells (Aungst et al., 2003) and periglomerular neurons (Fukunaga et al., 2014), play a key role in synchronizing the network at the theta frequency and would indirectly control the conditions necessary for the entrainment of the network at the gamma frequency (as the synchrony of MCs on the respiratory rhythm is necessary for the gamma emergence (Fig. 5*B*,*C*). Eventually non-GC neurons in the internal plexiform layer, like disynaptic short-axon cells (Pressler and Strowbridge, 2006; Eyre et al., 2008), could play a role that was not yet explored. Second, TCs were also not included in our model. TCs initiate the olfactory bulb response to the sensory stimulation (Fukunaga et al., 2012), but mostly have been hypothesized as being responsible for the high gamma oscillations (Manabe and Mori, 2013). Our model predicts that the gamma frequency depends on the average firing rate of the MC population. Then we could expect that when TCs get involved in the gamma rhythms, the gamma frequency increases. Similarly, an earlier respiration locking compared with MCs (Buonviso et al., 2003; Fukunaga et al., 2012) could explain the differential timing of the high gamma and slow gamma oscillations through the respiratory cycle (Manabe and Mori, 2013).

A third point to be justified is our choice of a phase model for the odor–concentration/odor–intensity dependence of the response of mitral cells. It is a general observation that the concentration effect on bulbar activity is complex and includes linear, but also nonlinear, effects on the network response. Among a number of studies, some showed a relatively proportional relation between firing rate and odor intensity (Mair, 1982; Cang and Isaacson, 2003) or a clear relationship between pattern and intensity (Harrison and Scott, 1986; Reinken and Schmidt, 1986; Wellis et al., 1989). Alternatively, Chalansonnet and Chaput (1998) showed that increasing odor concentration did not change the mean firing frequency of individual cells but tended to shift the respiratory phase of the cells. Others have reported pattern changes that cannot be predicted from the response to a particular intensity (Kauer, 1974; Meredith, 1986). We chose to simulate variations in odor intensity by MC spike phase locking, which seems to be one of the critical parameters varying with intensity (Margrie and Schaefer, 2003; Courtiol et al., 2011a; Fukunaga et al., 2012), as it also increases the chance of MCs and GCs to fire together during the respiratory cycle. Other MC response parameters that were shown experimentally to vary with odor intensity, such as the firing rate or the number of activated glomeruli, as has been reported in many studies (Meister and Bonhoeffer, 2001; Khan et al., 2010), were not directly studied here. Although the influence of the above parameters could explain the decrease in gamma oscillations, as reported in Figure 2, they cannot by themselves only explain the experimentally observed increase of the beta oscillations because the phase dispersion of MC firing is the critical parameter in our model for the emergence of beta oscillation. Other parameters not included in this model, like the fine balance regulating the tonic excitation and inhibition (Yokoi et al., 1995), could also alleviate those limitations.

Last, the crucial existence for the model of the respiratory phase-shifted and subthreshold centrifugal input for the generation of a beta oscillation during the expiration is debatable because only a few direct measurements of those inputs to the olfactory bulb were performed. Imaging techniques (Rothermel and Wachowiak, 2014) could solve this. In our case, this phase shift is necessary to explain the different phases of gamma and beta oscillations observed only under anesthesia, because in awake conditions a phase shift is not required in regard to the lack of alternation between gamma and beta oscillations during the course of a respiratory cycle.

Overall, we presented here the necessary and sufficient components of the network to, first, generate an entrainment of MC activities based a weak inhibition from GC to MC at the gamma frequency and, second, a synchronization of MCs based on a strong inhibition from GCs to MCs. Whether or not our results can be fully extended to the awake and functional conditions remains an open question. Existing data in the awake preparation shows that gamma oscillations are either increased (Martin et al., 2006) or decreased (Kay, 2003) by odor in a learning paradigm; other studies report either an enhancement (Zibrowski and Vanderwolf, 1997) or no modification (Beshel et al., 2007) in beta power. The only clear conclusion that can be drawn from all these studies is that the dynamics of beta and gamma oscillatory activity largely depends on the task and the behavioral strategy. We thus seem to have a good argument for postulating that the activity of the OB in the anesthetized animal, such as simulated in our model, reflects the “wiring-imposed” dynamics. On top of these basal dynamics, variations related to learning, attention, and/or expectation are likely to be superimposed in the behaving animal, resulting in variations around the classic beta/gamma alternation observed in the anesthetized preparation. Thus, the framework we propose in our model is likely to be recruited in awake conditions with some modalities that remain to be determined and could be added to the model to enrich its panel of responses.

### Competition between beta and gamma oscillations: model specificities and olfactory functions

A prominent aspect of OB *in vivo* beta and gamma oscillations is that they do not occur simultaneously, but alternatively. The mechanisms used in this model capture this behavior in two ways. First, when strong centrifugal input allows GCs to start spiking, the strong inhibition of MCs prevents them from oscillating at the gamma frequency. Second, when MCs are strongly activated by sensory inputs, the STD of MC–GC excitatory synapses (Balu et al., 2007) prevents MCs from activating GCs above their spiking threshold and thus preserves the gamma oscillation. For the latter, it is important that gamma oscillations can develop first, in order to trigger the STD process, which is made possible by the phase shift between sensory and centrifugal inputs at the respiration frequency.

Regarding gamma oscillation dynamics, our model proposes that they can emerge from weak coupling due to local activation of GCs. This result proposes an alternative to previous studies (Bathellier et al., 2006, 2008b) and gives further insight into the debate between coupling (Llinás, 1988) and inhibitory feedback (Freeman, 1975; Eeckman and Freeman, 1990) to generate gamma oscillation in the olfactory bulb as here both are present and compatible. Although the gamma frequency depends strongly on the intensity of sensory inputs, as in previous models, the gamma amplitude decreases strongly when it leaves the 60–80 Hz range (Fig. 2*I*). The lower bound of the gamma oscillation is linked to the minimal interspike interval set by the intrinsic bursting activity of MCs (namely, due to its slow potassium channel; Wang, 1993), and the upper bound is set by synaptic inhibitory feedback properties of the network that cannot function above a certain frequency. This maximum of amplitude in the gamma range can also be linked to the optimum of the entrainment susceptibility of MCs in the gamma frequency range (David et al., 2009). Overall, these mechanisms likely contribute to the gamma oscillation frequency boundaries in the OB, as has been observed experimentally (Buonviso et al., 2003; Ravel et al., 2003; Rojas-Líbano and Kay, 2008).

Regarding beta oscillations, we proposed that they occur through a PING mechanism that is possible only when GCs are in a state of high excitability. This high-excitability state can be induced by a slowly modulated and broad centrifugal excitatory input to GCs (Balu et al., 2007; Pressler et al., 2007; Matsutani and Yamamoto, 2008). Importantly, to stay in the PING regime, the centrifugal drive to GCs must remain subthreshold. This is supported by a recent experimental study (Boyd et al., 2012) that showed *in vivo* that broad OB centrifugal fiber activation did not affect OB spontaneous activity but increased odor-evoked recurrent excitation on MCs. In contrast, beta oscillations cease when centrifugal inputs are depressed (Neville and Haberly, 2003; Martin et al., 2006). These early studies suggested that beta oscillations could result from a long feedback loop between OB and piriform cortex. Instead, our model demonstrated that beta waves could be generated in isolated OBs and did not require a loop functioning at the beta frequency between the olfactory bulb and the piriform cortex, but rather required a slow modulation of the GC excitability by the piriform cortex. Other factors controlling the functioning of the GC–MC synapse, such as acetylcholine (Pressler et al., 2007) or norepinephrine (Mouly et al., 1995), should also have a strong impact on gamma or beta oscillation regimes. Their effects remain mostly described in animal behavioral performance (Fletcher and Chen, 2010).

Functionally, the influence of gamma and beta oscillations on discrimination and learning is still highly debated. If gamma oscillations were shown to be critically involved (Lepousez and Lledo, 2013), the role of beta oscillations in learning and discrimination is still unknown despite their strong correlation with olfactory tasks (Martin and Ravel, 2014). In mammals, the independent manipulation of gamma and beta oscillations has not been performed in behaving animals yet, so that their respective role remains unclear. Modeling the competition between the two dynamics could not only explain why gamma and beta oscillations can appear in various contexts and paradigms not necessarily linked to a similar meaning or function (but unified by a similar dynamic process), but in the future should also be able to associate the involved mechanisms with their role in defining the behavioral responses to odorants.

### Conclusion

Overall, our results show that the competition between gamma and beta oscillations depends on a mix of parameters including the nature, intensity, and valence of the odor, along with the sniffing strategy of the animal. Disentangling the origins of the mechanisms governing the switch would require a selective activation of the GCs to mimic the centrifugal feedback while monitoring the oscillatory regime of the olfactory bulb. Functionally, relating these mechanisms to recent studies that emphasize the roles of gamma and beta oscillations to transmit information in a, respectively, feedforward and feedback manner (Roelfsema, 2006; Bastos et al., 2015) could reveal the essential functions of these waves in the OB. In particular, the ability of a given structure to internally produce a specific rhythm depending on its major input can allow it to be more sensitive to input from other structures oscillating at the same frequency (Fries, 2005). Regarding the olfactory system, this could dynamically change the functional connectivity between the OB and the piriform cortex (Franks and Isaacson, 2005, 2006; Arenkiel et al., 2007; Oswald and Urban, 2012) or the accessory olfactory nucleus (Hagiwara et al., 2012). Additional modeling studies including such structures could be helpful to infer the information transmission as a function of the OB dynamic state, as well as connectivity analyses in multistructure recording experiments with a learning paradigm where beta oscillations increase across sessions.
